# Supplementary material for: Atomistic-Level Structural Insight into Vespa Venom (Ves a 1) and Lipid Membrane Through the View of Molecular Dynamics Simulation
Source: Toxins (Basel). 2025 Jul 31;17(8):387. doi: 10.3390/toxins17080387 (PMC12389835; doi:10.3390/toxins17080387)
Supplement: Supplementary file 1 [file toxins-17-00387-s001.zip › Figure S1.pdf]

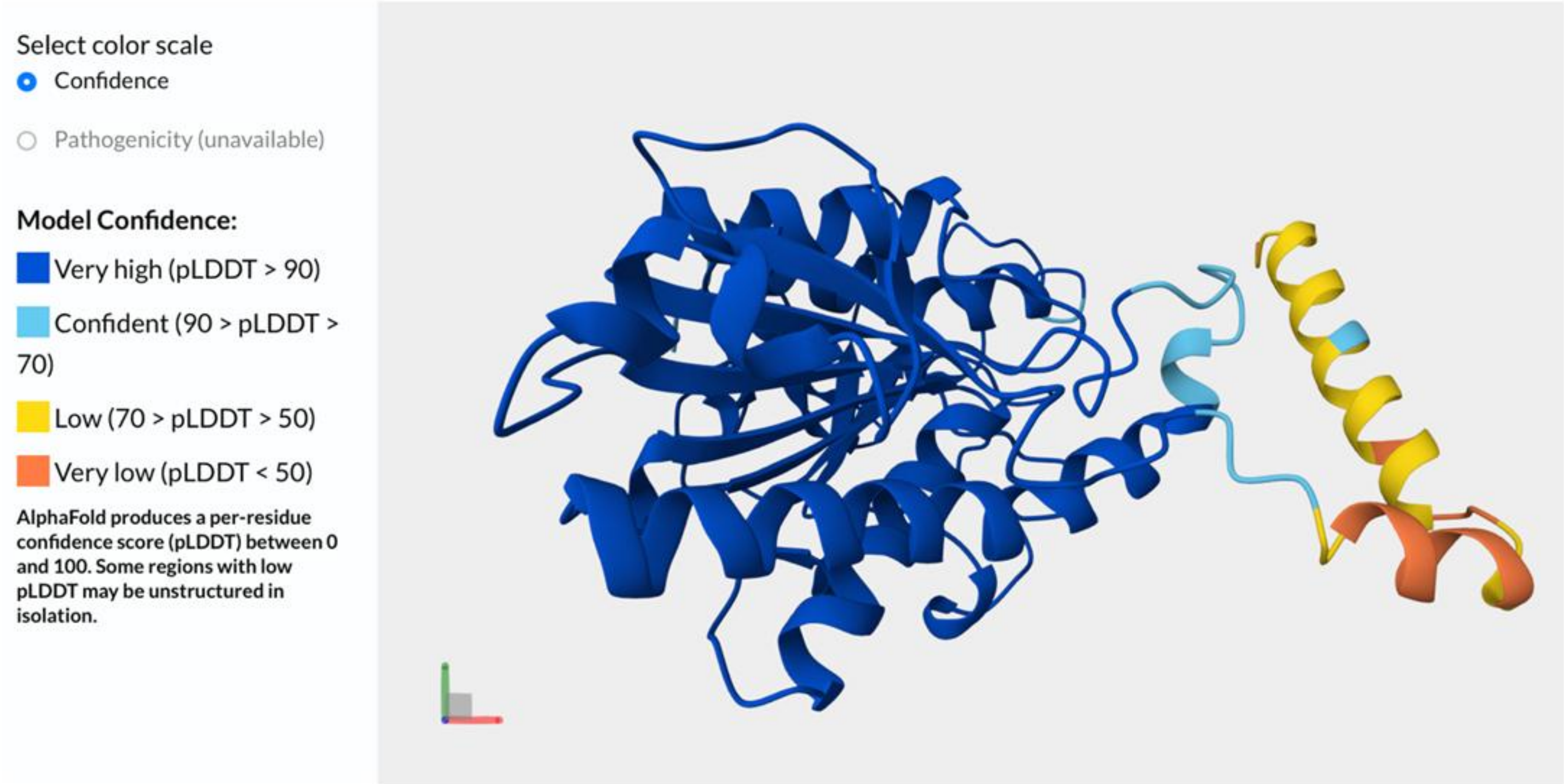

Figure S1. The 3D structure of Ves a 1 was predicted using AlphaFold based on the UniProt entry (ID: P0DMB4), with the corresponding AlphaFold model ID: AF-P0DMB4-F1-v4. The per-residue confidence score (pLDDT) indicated very high confidence (pLDDT > 90), shown in dark blue, particularly in the core regions of the structure used in this study.
